# Pure Mandibular Incisor Intrusion: A Finite Element Study to Evaluate the Segmented Arch Technique

**DOI:** 10.3390/ma12172784

**Published:** 2019-08-29

**Authors:** Gabriela Meyge de Brito, Hélio Henrique de Araújo Brito, Gabriel Goulart Mendes Marra, Laíze Rosa Pires Freitas, Bernardo Oliveira Hargreaves, Pedro Américo Almeida Magalhães, Dauro Douglas Oliveira

**Affiliations:** 1 Department of Dentistry, Graduate Program in Orthodontics, Pontifical Catholic University of Minas Gerais, Belo Horizonte 30535-901, Brazil; 2 Department of Mechanical Engineering, Pontifical Catholic University of Minas Gerais, Belo Horizonte 30535-901, Brazil

**Keywords:** finite element analysis, orthodontic tooth movement, mandibular incisors, intrusion

## Abstract

Leveling the curve of Spee is a commonly-used strategy to correct deep bites. Although several techniques have been proposed to intrude mandibular incisors (MI), flaring of these teeth is often observed and in many instances undesired. A three-dimensional (3D) finite element model (FEM) was used to locate the ideal point of force application (PFA) to achieve pure MI intrusion with the three-piece arches’ technique. It comprised (1) a 0.021 × 0.025 in. stainless steel (SS) wire that passively filled the slots of the canine and premolar brackets and the first and second molar tubes, bilaterally; (2) a 0.0215 × 0.0275 in. SS intrusion base arch (IBA) inserted into the MI brackets, that presented a step down distal to the lateral incisors brackets and a posterior extension arm; (3) titanium-molybdenum tip-back springs designed to apply the intrusion force, fitted inside the first molar gingival tube. Four PFA on the IBA were simulated (FEM 1, 2, 3, and 4). FEM 3 resulted in pure MI and was considered the ideal PFA. FEM1 and 2 showed intrusion and buccal crown flaring of the MI, whereas FEM4 resulted in intrusion and lingual crown flaring of those teeth. Clinicians may consider three-piece arch mechanics to achieve pure MI intrusion. However, they must be aware that when force was applied anteriorly or posteriorly to the ideal PFA, the incisors would incline labially or lingually, respectively.

## 1. Introduction

Leveling the curve of Spee is commonly used to correct dental deep bites [1,2,3,4,5]. Posterior teeth extrusion [6], mandibular incisors intrusion [7,8], or a combination of both tooth movements are often used to orthodontically level the curve of Spee [9,10,11,12]. Deep bites typically occur in association with Class II malocclusions [11,12,13]; thus, the extrusion of the posterior teeth may be detrimental because undesired clockwise mandibular rotation may occur [13] and worsen the sagittal relationship [2]. Although several techniques have been proposed to achieve pure mandibular incisor intrusion, flaring of these teeth is often observed [14,15,16,17].

In the 1970s, Burstone introduced the segmental arch technique to intrude the mandibular incisors and thus level the curve of Spee [2,18,19]. This clinical approach uses wires that do not run continuously from right to left molars. Burstone’s mechanism for intrusion consists of three parts: (1) a posterior anchorage unit that uses a buccal stabilizing segment of wire that is inserted into the posterior teeth brackets or tubes, bilaterally; (2) an anterior segment in the four incisors; and (3) intrusive arch springs, also bilaterally. The latter are inserted into the auxiliary gingival tube of the first molars and are activated with a tip-back bend, and when the spring is attached to the anterior segment wire, it delivers an intrusion force on the incisors. This author suggested that this approach is capable of achieving genuine intrusive movement of the incisors with minimum side effects. However, Weiland et al. [15] evaluated the efficacy of both segmented and continuous arch techniques for the correction of deep bites. These authors found significant proclination of the mandibular incisors with both mechanics. The major advantage of the segmented arch technique was the significantly smaller mandibular molar extrusion. Shroff et al. [20,21,22] updated Burstone´s segmented arch technique by changing the location of the point of force application to intrude the incisors. They estimated the center of resistance (CR) of the four incisors and used this information to establish the point of force application during intrusive mechanics. Despite the significant clinical relevance of these reports, no studies confirming this information have been reported.

Labial flaring of the mandibular incisors may increase the risk of developing gingival recession because these teeth may be pushed out of the alveolar bone process [23,24]. It may also lead to anterior occlusal trauma (fremitus), which has been listed as an etiological factor in the development of temporomandibular joint disorders [25]. Furthermore, the orthodontic treatment stability may be questionable when the mandibular incisors are labially inclined [18,26]. Despite the importance of this topic, studies in the orthodontic literature reporting mechanical systems that can truly achieve pure mandibular incisor intrusion are lacking.

The finite element (FE) analyses method uses mathematical calculations to evaluate the effects of external loads applied to three-dimensional (3D) structures. This method is suitable and reliable for the testing of the biomechanical effects of different mechanics in orthodontics [3,27,28,29,30], analyzing the stress and displacement of structures under various boundary and loading conditions and studying areas that are difficult or impossible to access without any risks to a human sample [31,32].

The purpose of this research was to use the FE method to simulate the effects of different extensions of the cantilevers that are used in three-piece segmented mechanics to level the curve of Spee.

## 2. Materials and Methods

### 2.1. Modeling

**Figure 1:**
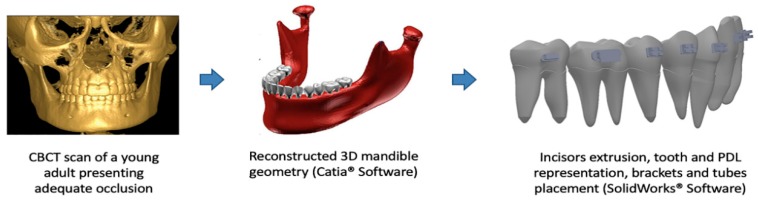
Sequence for three-dimensional (3D) model construction and finite element (FE) representation of tooth, periodontal ligaments PDL, and bone.

The 3D FE model construction was initiated with 2 mm sections of a computerized tomography (CT) scan (Picker, model PQ2000; Highlands Heights, OH, USA) of a young adult with complete permanent dentition, with the exception of the third molars, which exhibited normal occlusion. All of the two-dimensional CT scan images were stacked with computer design software (CATIA, Dassault Technologies, Woodland Hills, CA, USA) to accurately develop the model of the mandible. During the development of this model, the teeth were built independently and were considered as independent parts with uniform mechanical properties. This model was obtained from a previous study by our research group and was updated and improved [32]. The 14 teeth were modified until the proper crown-to-root ratio was obtained according to Wheleer [33], and the teeth were aligned according to the Peer Assessment Rating index [20]. The periodontal ligaments (PDLs) were modeled with 0.20 mm linear thicknesses, as previously described in other FE studies [27,30]. To represent an excessive curve of Spee, the four incisors were extruded 2.5 mm. All modifications to this initial FEM model were performed using SolidWorks Software (version 2019 SPO.0, Dassault Systèmes Americas Corp. Waltham, MA, USA) (Figure 1).

Graphic representations of the fixed orthodontic appliances were modeled with 0.022 × 0.028 in. slots and zero degrees of tip and torque, and were placed on the centers of the crowns of all mandibular teeth. The first molar tube was a 0.018 × 0.025 in. gingival tube, which is common among the majority of most double tubes that are available on the market.

**Figure 2:**
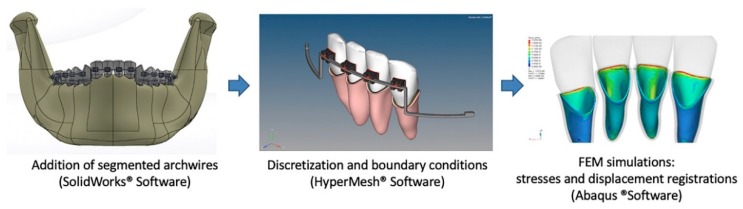
Sequence for segmented arch technique graphic representation and finite element model (FEM) discretization and simulation.

The three-piece segmented archwires comprised the following: (1) a 0.021 × 0.025 in. stainless steel (SS) base wire that bilaterally passively filled the second molar tube, the first molar main tube, the premolars, and the canine bracket slots to simulate the posterior anchorage segment (the posterior teeth were leveled and aligned to allow for the passive fit of these wires); (2) a 0.0215 × 0.0275 in. SS intrusion base arch (IBA) that was inserted into the incisor brackets and was also a passive wire that exhibited a step-down distal to the lateral incisor brackets and a 15 mm posterior extension arm below the canine and premolar brackets; and (3) a 0.017 × 0.025 in. titanium molybdenum (TMA) tip-back springs that were designed to apply the intrusion force. The distal ends of the cantilevers were fit inside the first molar gingival tubes, and a helix of 3 mm in diameter was constructed to be mesially flush to the tubes (Figure 2).

This helix was designed to simulate what we consider to be more clinically applicable, since in a clinical setting it is used to tie the cantilever to the molar tube and reduce the chances of becoming loose from the auxiliary tube during mastication or oral hygiene procedures.

### 2.2. Simulated Points of Force Application

**Figure 3:**
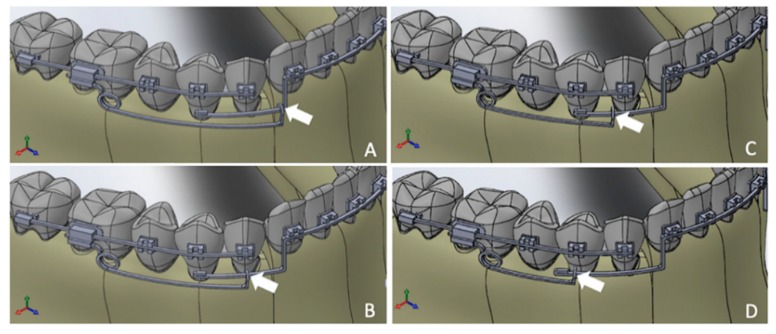
FEM tested: (**A**) FEM 1; (**B**) FEM 2; (**C**) FEM 3; (**D**) FEM 4. Arrows indicate the exact location of the points of force application evaluated.

Four different 3D FE models were constructed, and the only difference between these models was the mesiodistal length of their cantilevers. Therefore, four different points of force application on the IBA were tested: FEM 1 was the longest cantilever and was thus the most anterior point of force application on the IBA; FEM 2, in which the point of force application was located at the level of the mesiodistal center of the canine crown; FEM 3, in which the cantilever length was 2 mm shorter than that in FEM 2; and FEM 4, which involved the shortest cantilever and resulted in a point of force application at the level mesiodistal center of the first premolar crown (Figure 3).

All of these models included both the right and left sides; thus, the points of force application were bilaterally tested.

### 2.3. Discretization and Boundary Conditions

Following the model construction, the discretizations and identifications of the boundary conditions of the tested anatomical structures and materials were performed using HyperMesh Software (Altair-Engineering, Inc. Milwaukee, WI, USA). The FE models were considered to have linear elasticities and isometric properties. The PDLs and alveolar bones were hybrid meshes with pentahedron and hexahedron elements that provided a more accurate estimation of the stresses that occurred on these structures. The sophistication of the mesh was important because the PDLs were the structures in which the stresses were to be evaluated in the present research. The other objects contained pentahedron elements, and each element had six degrees of freedom; thus, these objects could move and rotate in any direction within the space. Finally, each pentahedron element had six nodes, and each hexahedron element had eight nodes. The Young moduli and Poisson ratios for the teeth, periodontal ligaments, brackets, tubes, and wires were defined as based on previous studies (Table 1) [25,27].

All bracket-wire contacts were set as rigid contacts; thus, no wire displacement within the brackets was possible.

The analyses were performed based on simulations of the activation of the cantilever to bilaterally deliver 20 gf [2] of intrusion force at four different points along the IBA. Thus, a total of 40 gf was applied to the incisors on the IBA by the cantilever in each simulation, and the anchorage teeth were under a reactive force of 20 gf, which generated a moment on the molar tube. That generated a couple that depended on the cantilever length. The use of the FE method allowed us to register the initial displacements and the von Mises stresses along the root surfaces of the four mandibular incisors and the posterior teeth that served as anchorage points. All of the simulations were performed using Abaqus Software (version 2019, Dassault Systèmes Americas Corp., Waltham, MA, USA).

## 3. Results

The total numbers of elements used in the FE models were 2,414,014, 2,407,131, 2,405,280, and 2,060,466 for FEM 1, FEM 2, FEM 3, and FEM 4, respectively. The von Mises stresses and the initial displacement tendency distributions in each model were descriptively analyzed using a color scale. Regarding the stress evaluations, colors corresponding to negative values indicated compression areas on the PDL, and positive values indicated tension areas. Regarding the initial displacement readings, the colors corresponding to negative values indicated posterior movement, and the positive values indicated anterior movement in the sagittal view.

### 3.1. Effects on the Mandibular Incisors

**Figure 4:**
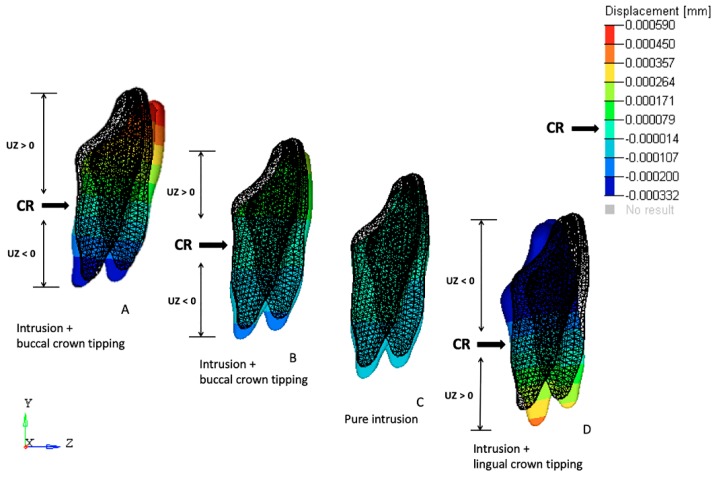
Initial displacement tendency of the mandibular incisors under 40 g of intrusive force in a sagittal view. Teeth displacement was magnified 500 times. Initial position (drawn) and final position (color). (**A**) FEM 1, (**B**) FEM 2, (**C**) FEM 3, and (**D**) FEM 4.

The initial displacement tendencies in FEM 1 were concentrated on the incisal edges and along the labial surfaces of all four incisors, suggesting that intrusion and buccal crown tipping would occur. Similar but less intense tendencies were registered in FEM 2. The FEM 3 simulations revealed uniform initial displacement tendency distributions on all surfaces of the four incisors, indicating intrusion without buccal or lingual crown tipping. Conversely, in FEM 4, the initial displacement tendencies were concentrated along the lingual surfaces of all of the incisors, suggesting tendencies toward intrusion and lingual crown tipping (Figure 4).

**Figure 5:**
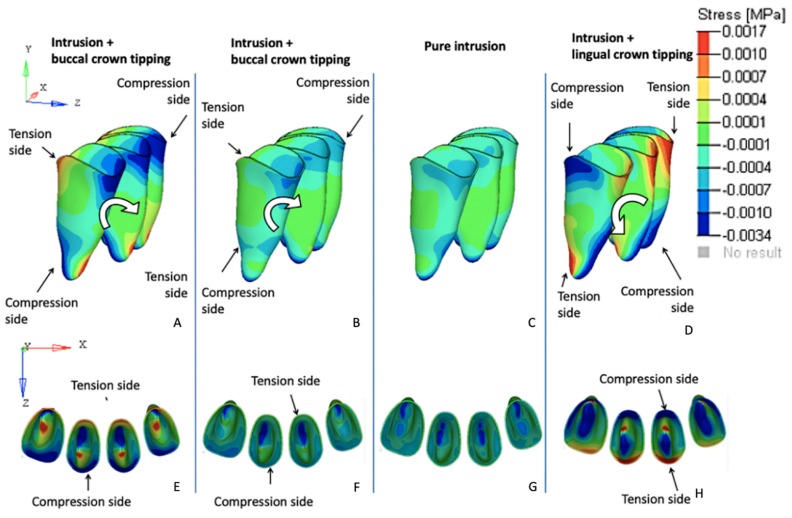
Von Misses stress of the mandibular incisors under 40 g of intrusive force in a sagittal (**A**, **B**, **C** and **D**) and occlusal (**E**, **F**, **G** and **H**) views. (**A**) and (**E**) FEM 1, (**B**) and (**F**) FEM 2, (**C**) and (**G**) FEM 3, and (**D**) and (**H**) FEM 4.

The von Mises stress tendencies registered for each variable complemented the results of the initial displacement tendencies described above; in both FEM 1 and FEM 2, the maximum stress values were concentrated on the buccal surfaces and on the apexes of the incisor roots. The FEM 3 simulation revealed that the amount of stress registered on the PDL of the incisors was uniformly distributed along the buccal and lingual surfaces and on the root apex. Finally, in FEM 4, the von Mises stresses were concentrated on the apexes and lingual surfaces (Figure 5).

**Figure 6:**
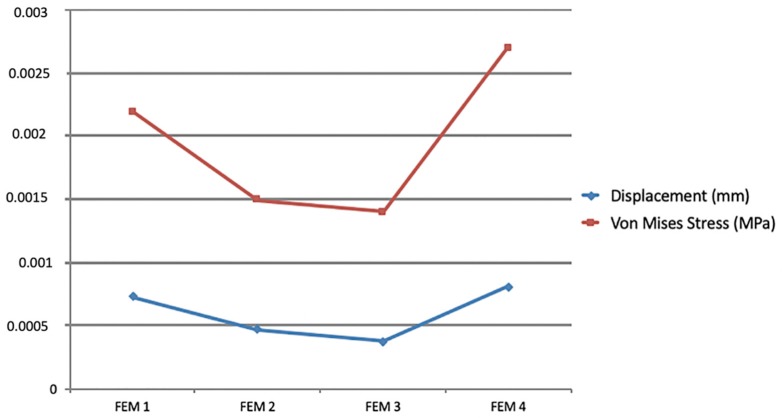
Maximum values for von Mises stress (**red**) and initial displacement (**blue**) for each variable tested (FEM 1, FEM 2, FEM 3, and FEM 4) on anterior teeth.

The maximum von Mises stress and initial displacement tendency values are shown in Figure 6 for each of the simulated FEM models.

### 3.2. Effects on the Posterior Anchorage Segment

The stresses and initial displacement tendencies produced in the PDL of the posterior teeth that were used as dental anchorage points occurred primarily in the first molars. These teeth exhibited displacement tendencies that were compatible with a tip-back movement tendency (Figure 6).

**Figure 7:**
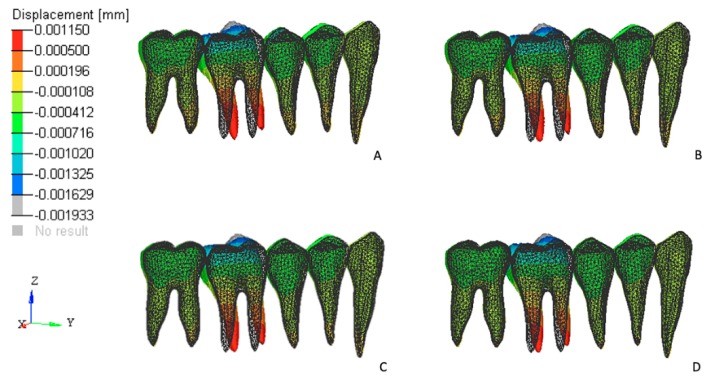
Initial displacement tendencies on the teeth that served as the posterior anchorage unit from a sagittal view. Teeth displacement was magnified 3000 times. Initial position (drawn) and final position (color). (**A**) FEM 1, (**B**) FEM 2, (**C**) FEM 3, and (**D**) FEM 4.

FEM 1 exhibited the highest values for both the von Mises stresses and initial displacements. These values progressively decreased in all other models as the tested cantilevers became shorter (in the FEM 2, FEM 3, and FEM 4 models; Figure 7).

**Figure 8:**
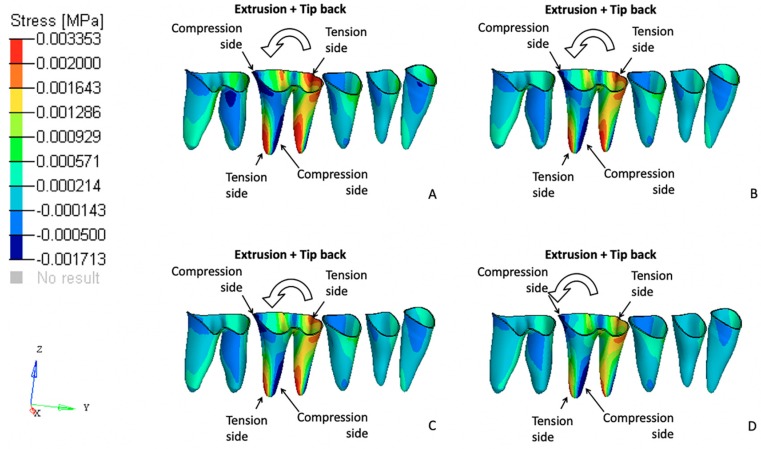
Von Mises stress on the teeth that served as the posterior anchorage unit from a sagittal view. (**A**) FEM 1, (**B**) FEM 2, (**C**) FEM 3, and (**D**) FEM 4.

The maximum von Mises stress and initial displacement values are shown in Figure 8.

The greatest displacement values were registered in FEM 1 (2.067 × 10^−3^ mm), and the maximum values also decreased as shorter cantilevers were simulated.

## 4. Discussion

The present FE study evaluated four different locations for the application of intrusive forces in the IBA to identify the ideal point of force application to achieve pure intrusion of all mandibular incisors. In this research, Shroff’s segmented arch technique was simulated to promote only the intrusion of the four mandibular incisors. The simultaneous intrusion and retraction of these teeth were not tested. The simulations revealed that pure mandibular incisor intrusion without labial or lingual inclination could be achieved when the ideal point of force application was used.

We found that the ideal point of force application on the IBA was located 2 mm distal to the mesiodistal center of the canines. Therefore, FEM 3 simulations exhibited pure intrusion without labial or lingual incisor inclination tendencies. From a biomechanical perspective, these results may suggest that the intrusive force generated in FEM 3 passed through the estimated position of the CR of the four mandibular incisors in this individual model. Our results may serve as a good reference for clinical orthodontists to begin the intrusion of mandibular incisors. However, individual patient variations, such as different sizes of the incisors, their initial labio-lingual inclinations, and/or alveolar bone levels, may alter the ideal point of force application on the IBA by slightly moving it anteriorly or posteriorly. We consider these variables of great importance and our research group will test them in a further experiment. As registered in FEM 1 and FEM 4, if proclination or retraction of the four mandibular incisors are required, the clinician may adjust the length of the cantilevers to apply the intrusive force more anteriorly or posteriorly, respectively.

The FE model evaluated in this study presented both mandibular canines as part of the posterior anchorage segments. The posterior teeth were leveled and aligned prior to the incisors’ intrusion, as previously recommended [12]. If we simulated the simultaneous intrusion of all six anterior teeth, the force required would be excessive and a large movement would be registered at the posterior teeth. Thus, the tip-back movement of the posterior teeth would occur more rapidly than the intrusion of the anterior teeth. The second important reason for this sequence is the incorporation of the canines into the posterior anchorage segments to reinforce it. The results of this study revealed that the von Mises stresses were distributed on all teeth of the posterior anchorage segment. However, a concentration of stress on the first molars was observed.

Mini-screws have been used to reinforce anchorage points and to minimize undesired side effects during deep bite corrections [34,35,36,37]. Senısık et al. [38] performed a clinical prospective study that compared the effects of mini-screws and Burstone´s intrusion arches in the intrusion of maxillary incisors while treating deep bite patients. The results found by these authors indicated that both mechanics led to intrusion but also induced proclination of the incisors. The maxillary molars in the intrusion arch group exhibited extrusion and distal crown tipping. The use of mini-screws eliminated these side effects. However, mini-screws represent an additional cost that is important to some patients and a surgical intervention. We observed similar effects on the anchorage teeth in our FEM simulations. However, the initial displacements observed were as low as 2.067 × 10^−3^ and distributed to all of the anchorage teeth. These results may suggest that no clinical molar extrusion would be observed when the three-piece segmented mechanic is used to intrude the mandibular incisors.

Intrusion is defined as the apical movement of the geometric center of the root in relation to the occlusal plane or to a plane based on the long axis of the tooth [2]. Although the tipping of the incisors may correct a deep bite, it actually produces a pseudo-intrusion of these teeth. The importance of obtaining pure mandibular incisor intrusion has been related to the prevention of fremitus [25], a reduction in the risk of developing periodontal attachment loss [23], and an increase in orthodontic treatment stability [39]. Furthermore, in Class II patients, true mandibular incisor intrusion would prevent the proclination of these teeth while leaving sufficient overjet to allow for adequate retraction of the maxillary anterior teeth.

Another important issue faced by the majority of orthodontists when treating patients who require intrusion is apical root resorption. Chiqueto et al. [40] evaluated the effects of accentuated and reversed curve of Spee mechanics on the root resorption of the incisors and found that this intrusion approach resulted in greater root resorption than that observed in the control group. Conversely, Costopulos and Nanda [41] found that intrusion caused only a negligible amount of root resorption. The main difference between these studies was the amount of force applied to intrude the incisors. The reversed and accentuated curve of Spee mechanics delivered 100 to 150 g of intrusive forces, and the Burstone´s intrusion arch delivered only 15 g per maxillary incisor. Our FEM study used 40 g to intrude all four incisors, and the results revealed that when pure intrusion was achieved (FEM 3), the lowest maximum von Mises stress values were observed (Figure 6). Because the same amount of force was applied in all of the simulated models, it can be stated that in FEM 3, the stress was homogeneously distributed over the root surfaces of the four teeth, whereas all of the other models exhibited stress that was concentrated in smaller areas either on the labial or lingual surfaces. Although FEM does not allow for the assessment of root resorption, based on the etiologies of root resorption, both the low forces and homogeneous stresses generated when true intrusion was obtained may reduce the chances of developing root resorption of the incisors.

Regarding the anchorage segment, the maximum von Mises stress and displacement tendency values decreased progressively as the cantilever length was shortened. This observation is due to the reduced moment generated as the distance to the point contact of the cantilever end was diminished (moment = force × distance; Figure 8). In this mechanic system, the cantilever represented a one-couple orthodontic appliance [42] in which one attachment generated a couple and a force on the auxiliary molar tube, and other attachment only produced a force (on the IBA). For this reason, the color scale results for the FEM simulations on the anchorage segment exhibited greater von Mises stresses on and initial displacements of those teeth compared with the incisors. Although the anchorage teeth stress and displacement values were clinically low, we may also expect that the masticatory forces on the posterior teeth may counteract the extrusion and tip-back tendencies of those teeth [2]. Differently, the cantilever length on the anterior teeth demonstrated a different behavior; a moment was generated only on models FEM 1 and FEM 2 (intrusion and buccal crow tipping) and on FEM 4 (intrusion and lingual crown tipping). FEM 3 showed pure intrusion, thus no moment was generated. Even though all four points of force application were located distal to the four incisors, lingual crown tipping was obtained only in FEM 4, which suggests that among the variables tested, this was the only one located distal to the CR of the four teeth. Although both FEM 1 and FEM 2 demonstrated buccal crown tipping, the resulting images showed a higher initial displacement and von Misses stress on FEM 1, as seen on the color scale (Figure 5). This can be explained by the higher movement generated in this model, since this point of force application is located further away from the CR.

Finite element simulations represent a suitable method for assessing the displacements and stress distributions of bodies that are exposed to stress. This study used static finite element analyses, which only simulate the initial tooth movement and stress distribution in the periodontal ligament. While the teeth move, the force systems are modified, and mechanical adjustments must be made during treatment to achieve the expected results.

## 5. Conclusions

1 The FEM simulations indicated that pure mandibular incisor intrusion was registered when the point of force application on IBA was 2 mm distal to the center of the canine crown in this individual model. Intrusive forces applied mesially to this reference point generated labial crown tipping tendencies while forces applied more than 2 mm distally to the center of the canine resulted in lingual crown tipping of the mandibular incisors.
2 The majority of the reaction forces registered on the posterior segments were concentrated on the first molar, and their effects were reduced when compared with the adjacent teeth on the anchorage unit.

## Figures and Tables

**Table 1:** Properties of anatomic structures and material tested. PDL = periodontal ligaments, SS = stainless steel.

	Elastic Modulus (E) (Mpa)	Poisson‘s Ratio (v)
Tooth	20.000	0.30
PDL	0.71	0.40
Bone	345	0.30
SS	200.000	0.30
TMA	69.000	0.30
